# Supplementary material for: The interactome of intact mitochondria by cross-linking mass spectrometry provides evidence for coexisting respiratory supercomplexes
Source: Mol Cell Proteomics. 2017 Dec 8;17(2):216–32. doi: 10.1074/mcp.RA117.000470 (PMC5795388; doi:10.1074/mcp.RA117.000470)
Supplement: Supplemental Data [file supp_RA117.000470_133922_0_supp_23978_fzffwf.zip › spectra_annotation/mito_DR_spectra_annotation/33-1-4-1-7-1.pdf]

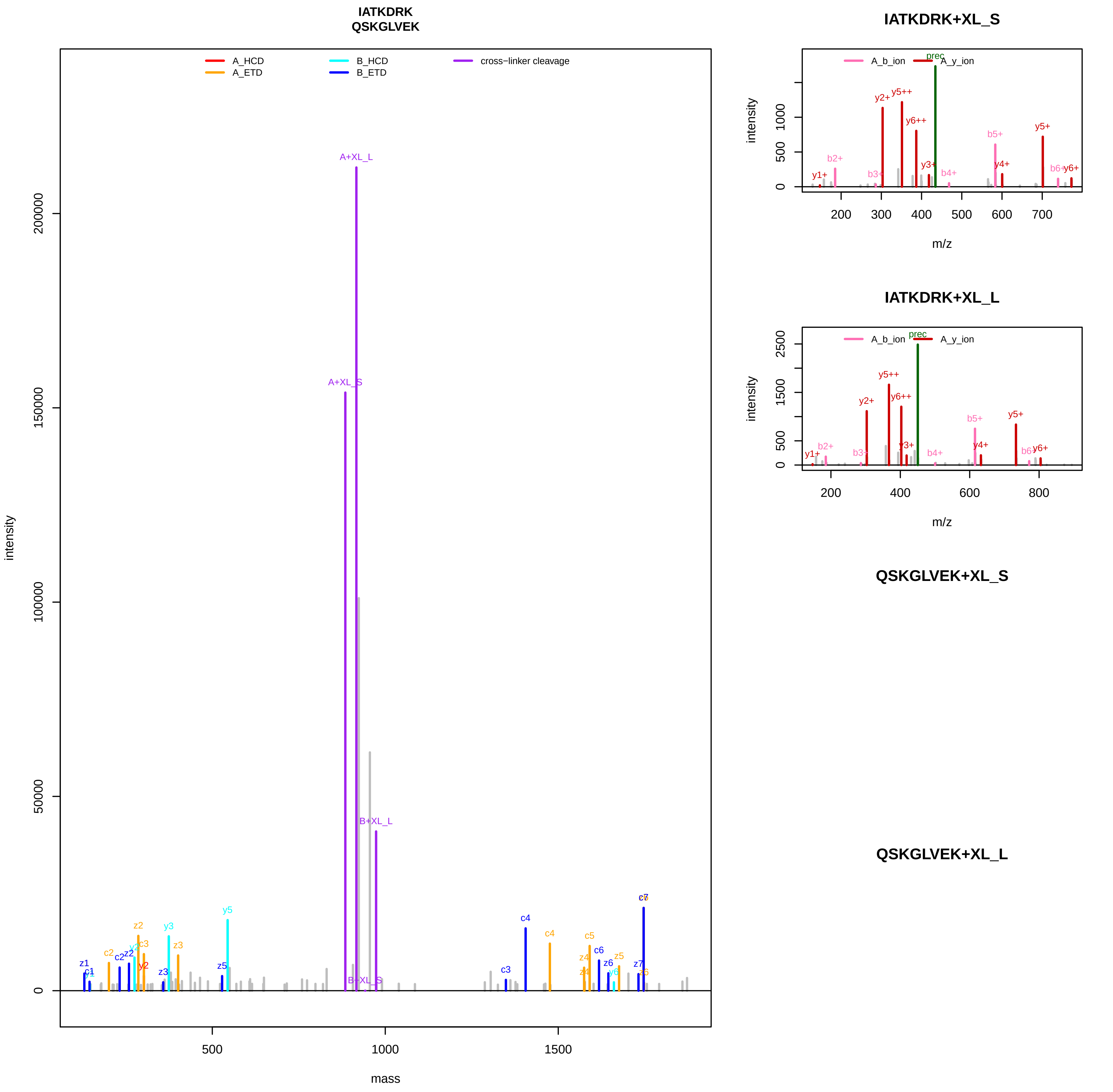
IATKDRK
QSKGLVEK
A_HCD
A_ETD
B_HCD
B_ETD
cross−linker cleavage
A+XL_L
A+XL_S
B+XL_L
B+XL_S
intensity
mass
0
50000
100000
150000
200000
500
1000
1500
z1
c1
c2
c2
z2
y2
z2
c3
y2
y3
z3
z3
y5
z5
c3
c4
c4
c5
z4
c6
z6
z5
y6
z7
c7
IATKDRK+XL_S
A_b_ion
A_y_ion
prec
y1+
b2+
b3+
y2+
y5++
y6++
y3+
b4+
b5+
y4+
y5+
b6+
y6+
m/z
200
300
400
500
600
700
1000
IATKDRK+XL_L
2500
1500
800
QSKGLVEK+XL_S
QSKGLVEK+XL_L
